# Molecular Analysis of the Prostacyclin Receptor’s Interaction with the PDZ1 Domain of Its Adaptor Protein PDZK1

**DOI:** 10.1371/journal.pone.0053819

**Published:** 2013-02-06

**Authors:** Gabriel Birrane, Eamon P. Mulvaney, Rinku Pal, B. Therese Kinsella, Olivier Kocher

**Affiliations:** 1 Division of Experimental Medicine, Beth Israel Deaconess Medical Center, Harvard Medical School, Boston, Massachusetts, United States of America; 2 School of Biomolecular and Biomedical Sciences, Conway Institute of Biomolecular and Biomedical Research, University College Dublin, Belfield, Dublin, Ireland; 3 Department of Pathology and Center for Vascular Biology Research, Beth Israel Deaconess Medical Center, Harvard Medical School, Boston, Massachusetts, United States of America; Russian Academy of Sciences, Institute for Biological Instrumentation, Russian Federation

## Abstract

The prostanoid prostacyclin, or prostaglandin I_2_, plays an essential role in many aspects of cardiovascular disease. The actions of prostacyclin are mainly mediated through its activation of the prostacyclin receptor or, in short, the IP. In recent studies, the cytoplasmic carboxy-terminal domain of the IP was shown to bind several PDZ domains of the multi-PDZ adaptor PDZK1. The interaction between the two proteins was found to enhance cell surface expression of the IP and to be functionally important in promoting prostacyclin-induced endothelial cell migration and angiogenesis. To investigate the interaction of the IP with the first PDZ domain (PDZ1) of PDZK1, we generated a nine residue peptide (KK^411^IAACSLC^417^) containing the seven carboxy-terminal amino acids of the IP and measured its binding affinity to a recombinant protein corresponding to PDZ1 by isothermal titration calorimetry. We determined that the IP interacts with PDZ1 with a binding affinity of 8.2 µM. Using the same technique, we also determined that the farnesylated form of carboxy-terminus of the IP does not bind to PDZ1. To understand the molecular basis of these findings, we solved the high resolution crystal structure of PDZ1 bound to a 7-residue peptide derived from the carboxy-terminus of the non-farnesylated form of IP (^411^IAACSLC^417^). Analysis of the structure demonstrates a critical role for the three carboxy-terminal amino acids in establishing a strong interaction with PDZ1 and explains the inability of the farnesylated form of IP to interact with the PDZ1 domain of PDZK1 at least *in vitro*.

## Introduction

Scaffold or adaptor proteins play key roles in facilitating and integrating signal transduction in multicellular organisms. These proteins recruit and/or anchor their binding partners to a specific subcellular location, serving as a platform to mediate or regulate interactions between proteins involved in diverse signalling pathways [1], [2], [3]. Typically, these proteins are modular by nature, consisting of a number of protein-protein interaction domains that bind target sequence(s) within their binding partner [1]. One of the most common protein-protein interaction motifs in scaffold or adaptor proteins is the PDZ domain, named from the first proteins identified to contain the motif, namely Postsynaptic density protein 95 (PSD-95), *Drosophila* Disks large tumour suppressor 1 (Dlg1), and Zonula occludens protein 1 (ZO-1). The PDZ domain typically spans approximately 80–90 residues and, structurally, is composed of six anti-parallel β-strands (βA–βF or β1–β6) sandwiched between two α-helices (αA/αB or α1/α2), with a highly conserved ‘GLGF sequence motif’ forming a hydrophobic binding pocket [4]. Typically, but not exclusively, PDZ domains bind a short region within the carboxyl (C-) terminus of their binding partner, a region termed the ‘PDZ ligand’ [5], [6]. The hydrophobic binding pocket and residues within neighbouring structural elements of a given PDZ domain are responsible for the sequence-specific recognition of the PDZ ligand within the binding partner [5].

PDZK1 is a member of the Na^+^/H^+^ exchanger regulatory family (NHERF) and is predominantly expressed in proximal epithelial cells of the kidney and in hepatocytes, and at a lower level in other epithelial cells and endothelial cells [7], [8], [9]. PDZK1 is a multi-PDZ domain containing protein, possessing four PDZ domains [7]. These domains facilitate the interaction of PDZK1 with a range of binding partners, including ion transporters (e.g. the cystic fibrosis transmembrane conductance regulator (CFTR) and apical organic cation transporters OCTN1 and OCTN2) and several members of the G protein-coupled receptor (GPCR) superfamily (e.g. the serotonin receptor 2B (HTR2B) and all five members of the somatostatin receptor (SSTR) family) [10], [11], [12], [13], [14]. In addition, through its interaction with the high density lipoprotein (HDL) scavenger receptor class B, type I (SR-BI), PDZK1 is essential for both reverse cholesterol transport (RCT) and for HDL-mediated vascular re-endothelialisation [8], [15].

The prostanoid prostacyclin, or prostaglandin (PG) I_2_, plays a central role in haemostasis, acting as a potent inhibitor of platelet aggregation and as an endothelium-derived vasodilator [16], [17]. It also exerts an important cytoprotective role within the myocardium [18] and, within the wider vasculature, promotes angiogenesis and limits restenosis enhancing re-endothelialisation/vascular repair in response to injury [17]. Prostacyclin primarily acts through the prostacyclin receptor or, in short, the IP, a member of the GPCR superfamily. PDZK1 was recently identified as a novel direct interactant of the IP and this interaction was determined to involve a Class I ‘PDZ ligand’ (Ser/Thr–X–Ф–COOH, where X represents any amino acid and Ф represents a hydrophobic amino acid) located at the extreme carboxyl terminus (-SLC^386^) of the human (h) IP [19]. Mutation of the P_0_ position (Cys^386^ (carboxy terminal end of the protein)) or the P_-2_ position (Ser^384^) of the PDZ ligand within the human (h)IP disrupted its interaction with PDZK1. Furthermore, mutation of the ‘GLGF motifs’ within the hydrophobic binding pockets of the four PDZ domains of PDZK1 suggested that the IP can bind to PDZ domain 1 (PDZ1), PDZ3 and PDZ4, but not PDZ2, of PDZK1 [19]. While PDZK1 did not influence overall levels of the IP, it was found to increase maturation of the IP at the plasma membrane and to be functionally important in promoting prostacyclin-induced endothelialisation and angiogenesis *in vitro* [19].

The IP is unusual among GPCRs in that it undergoes isoprenylation or, more specifically, farnesylation within an evolutionarily-conserved *CaaX* motif (-CSLC) at its extreme carboxyl terminal cytoplasmic domain [20], [21]. Classically, this lipid modification of *CaaX*-containing proteins involves the initial isoprenylation of the target Cys through carbon (C)-15 farnesylation or C-20 geranylgeranylation followed by proteolytic cleavage, or –*aaX*ing, of the three terminal residues and subsequent carboxy-methylation of the nascent terminal isoprenylated-Cys residue to form the fully processed farnesyl/geranylgeranyl-Cys-carboxymethyl ester [22], [23]. In the case of the IP, isoprenylation has been established to be critical for its efficient G protein coupling, effector signalling, and agonist-induced internalisation of the IP [20], [21], [24], [25], [26]. As stated, the discovery of the interaction of the IP with PDZK1 suggests that its *CaaX* motif (-CSLC) sequence may also serve as a ‘PDZ ligand’ and previous investigations have indicated that the interaction of PDZK1 with the IP is largely independent of its isoprenylation status [19]. However, the precise nature of the interaction of PDZK1, or of its individual PDZ domains, with the C-terminal region of the IP is not well understood and remains to be investigated at the molecular level.

Herein, we have further characterized the interaction between the IP and PDZK1. Isothermal titration calorimetry (ITC) was used to measure the binding affinity of IP-derived synthetic peptides to a recombinant protein corresponding to the first PDZ domain (PDZ1) of PDZK1. In addition, we determined the high resolution structure of PDZ1 bound to the ‘PDZ ligand’ of the IP by X-ray crystallography. We show that while the non-prenylated form of the IP is capable of interacting with PDZ1 *in vitro* with high affinity, the isoprenylated form of the receptor does not under the conditions tested.

## Materials and Methods

### Isothermal Titration Calorimetry (ITC)

**Figure 1:**
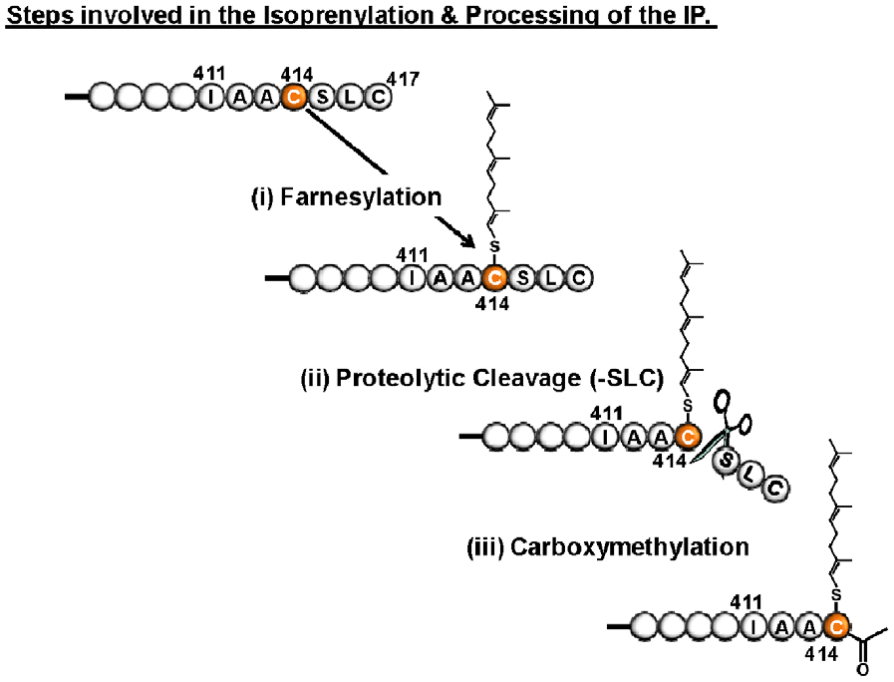
Steps involved in the isoprenylation and processing of the Prostacyclin Receptor. The prostacyclin receptor (IP) contains an evolutionary conserved ‘*CaaX* motif’ at its cytoplasmic carboxy-terminus, *e.g* corresponding to C^414^SLC^417^ of the mouse IP as shown. During its processing, (i) the IP undergoes isoprenylation through *thio-ether* attachment of a carbon (C)-15 farnesyl moiety to Cys^414^ while subsequent (ii) proteolytic cleavage, or *aaX*ing, liberates the terminal ^415^ *SLC* ^417^ residues and (iii) end-stage carboxy-methylation of the nascent α-carboxy-group on Cys^414^ generates the fully processed, mature IP in its farnesyl-Cys-carboxymethylated form. Herein, the interaction of peptides based on the mouse IP carboxy-terminus with PDZ domain 1 (PDZ1) or full length PDZK1 was investigated through isothermal titration calorimetry (ITC) where Peptide 1 is a nanopeptide containing the seven carboxy-terminal amino acids (KK^411^IAACSLC^417^); Peptide 2 is an octapeptide corresponding to the carboxy-terminus of IP (K^407^SEAIAAC^414^) devoid of the 3 terminal amino acids (-*aaX*/-^415^ *SLC* ^417^) which are proteolytically cleavage following farnesylation of the IP; Peptide 3 is identical to Peptide 2 (K^407^SEAIAAC^414^) except that it was modified by the addition of a C-15 farnesyl group on the carboxy-terminal cysteine (Cys^414^) and a carboxy-methyl group on the terminal -α-COOH, thereby representing the farnesyl-Cys-carboxymethyl ester form of the C-terminus of the IP.

Expression and purification of recombinant proteins corresponding to PDZ1 domain 1(PDZ1) of PDZK1 and to full length PDZK1 has been described previously [27]. Binding of synthetic peptides based on the C-terminus of the IP (Fig. 1) was measured using a VP-ITC microcalorimeter (GE Healthcare). Three individual peptides were used: Peptide 1 corresponded to the seven carboxy-terminal amino acids of mouse (m) IP, to which two N-terminal lysines (not part of the IP protein sequence) were added to increase peptide solubility (KK^411^IAACSLC^417^, ‘target peptide’); Peptide 2 consisted of an octapeptide corresponding to the carboxy-terminus of IP (K^407^SEAIAAC^414^) devoid of the 3 terminal amino acids (-aaX/-^415^SLC^417^) which would be removed by proteolytic cleavage following farnesylation [19]. The amino acid sequence of Peptide 3 was identical to that of the Peptide 2 (K^407^SEAIAAC^414^) except that it was modified by the addition of a C-15 farnesyl group on the carboxy-terminal cysteine (C^414^) and a carboxy-methyl group on the terminal –COOH, thereby representing the farnesyl-Cys-carboxymethyl ester form of the IP C-terminus. Peptides 1 and 2 were synthesized and HPLC purified by the Tufts University Core Facility (Boston, MA). Peptide 3 was synthesized by Thinkpeptides (Oxford, UK) and solubilized in DMSO. Briefly, 1.0 mM of the target peptides 1–3 was titrated against recombinant protein corresponding to PDZ1 or full length PDZK1 at concentrations of 0.03 mM in a buffer containing 150 mM NaCl, 25 mM Tris-pH 8.0 at 20°C under reducing conditions (0.5 mM tris (2-carboxyethyl) phosphine (TCEP)). Titration curves were analyzed and K_d_ values determined using ORIGIN 7.0 software (Origin Lab) with baseline correction. ITC experiments were performed in triplicate when binding of the peptide to the recombinant proteins was observed (peptide 1) and in duplicate when no binding was seen (peptides 2 and 3).

### Crystallography

A DNA fragment encoding the chimeric PDZ1–IP recombinant protein (residues 7–106 of PDZK1 - including PDZ1 (residues 7–86), 20 residues from the region of the protein between the PDZ1 and PDZ2 domains (interdomain residues 87–106) and, fused to the C-terminus of this interdomain segment, the seven carboxy-terminal amino acids of mouse IP (^411^IAACSLC^417^), was cloned into the pGEX-4T-3 vector and expressed in *E. coli* JM109 cells to produce a glutathione-S-transferase fusion protein. The recombinant protein was purified on glutathione-Sepharose 4B, released from GST using thrombin digestion and further purified by FPLC using a Superdex S75 column (GE Healthcare). The isolated, recombinant protein (108 residues total) contains an additional two residues (Gly-Ser, from the cloning vector) at the N-terminus that are not normally present in PDZK1. After initial screening for optimal crystallization conditions using the PEG suite (Qiagen), the PDZ1–IP chimera at 1 mM concentration was crystallized by the sitting drop vapor diffusion method at 18°C in a well containing 0.1 M MES pH 6.5 and 30% (w/v) PEG 300. Crystals were flash frozen directly into liquid nitrogen. Diffraction data were collected on beamline X29 at the National Synchrotron Light Source (Brookhaven National Laboratory, NY). The crystals belong to space group P2_1_ with unit cell dimensions of a = 39.270, b = 68.477, c = 40.124 Å with β = 91.8°. The data were reduced and merged using the HKL2000 suite [28]. Data collection and processing statistics are given in Table 1.

**Table 1:** Structure determination and refinement statistics.

Data collection	
Wavelength (Å)	1.075
Resolution^a^ (Å)	100.0-1.70 (1.76–1.70)
Completeness (%)	99.9 (99.8)
Total observations	159,150
Unique observations	23,390 (2,342)
Redundancy	6.8 (6.1)
R_sym_	9.6 (76.9)
<I/σ(I)>	17.4 (2.3)
**Refinement**	
Resolution (Å)	40.1–1.70
R_cryst_ (%)	18.4
R_free_ (%)	21.9
rmsd bond lengths (Å)	0.013
rmsd bond angles (°)	1.591
Ramachandran plot	
Preferred/Allowed/Outliers (%)	99.6/0.4/0.0

a Values in parenthesis are for the highest resolution shell. R_sym_ = Σ|I-<I>|/Σ(I), where I is the observed integrated intensity, <I> is the average integrated intensity obtained from multiple measurements, and the summation is over all observable reflections. R_cryst_ = Σ||F_obs_|-*k*|F_calc_||/Σ|F_obs_|, where F_obs_ and F_calc_ are the observed and calculates structure factors, respectively. R_free_ is calculated as R_cryst_ using 5% of the reflection chosen randomly and omitted from the refinement calculations. Bond lengths and angles are root-mean-square deviations from ideal values.

### Structure Determination and Refinement

The PDZ1-IP chimeric structure was solved by molecular replacement with PHASER [29] using the coordinates of PDZ1-SR-BI structure (Protein Data Bank identification code: 3NGH) as a search model. Refinement was carried out with REFMAC5 [30] and model building and addition of water molecules was performed manually using Coot [31]. The atomic coordinates and structure factors have been deposited in the Protein Data Bank as entry 4F8K.

## Results

We recently reported the functional interaction of the prostacyclin receptor (IP) with several PDZ domains of PDZK1, including PDZ1, PDZ3 and PDZ4 [19]. In order to determine the molecular nature of this interaction, we used biophysical techniques including isothermal titration calorimetry (ITC) and X-ray crystallography to analyze the binding of synthetic peptides based on the C-terminal region of the IP with recombinant proteins corresponding to full length PDZK1 or to its isolated PDZ1 domain.

### Isothermal Titration Calorimetry (ITC)

Initially, we performed ITC experiments using three synthetic peptides based on the C-terminal region of the IP (Fig. 1). The first peptide corresponded to the seven carboxy-terminal amino acids of mouse IP to which two lysines were added to the amino terminus to increase peptide solubility (KK^411^IAACSLC^417^). The second peptide (K^407^SEAIAAC^414^) corresponded to the carboxy-terminal sequence of the IP, but did not include the most carboxy-terminal three amino acids (-^415^SLC^417^) which are classically proteolytically cleaved (-*aaX*ed) in the mature IP following the initial isoprenylation step (Fig. 1). The third peptide contained the identical amino acid sequence of the second peptide (K^407^SEAIAAC^414^), except that the ^414^Cys was modified by the addition of both a farnesyl and a carboxymethyl group, thereby representing the mature, fully processed farnesyl-Cys-carboxymethyl ester form of the IP (Fig. 1). The rational for the use of peptides 1 and 3 was to investigate and compare the binding characteristics of the immature, unprocessed IP C-terminal sequence (peptide 1) relative to that of the mature, isoprenylated and proteolytically processed IP sequence (peptide 3) to PDZ1 or to full length PDZK1. While the sequence corresponding to peptide 2 may not be physiologically relevant, as it would not be found associated with the IP in nature, by comparing the binding characteristics of peptides 2 and 3, it was sought to determine the role or contribution of the farnesyl-group *per se* in the interaction of the IP with PDZ1 or with full length PDZK1.

**Figure 2:**
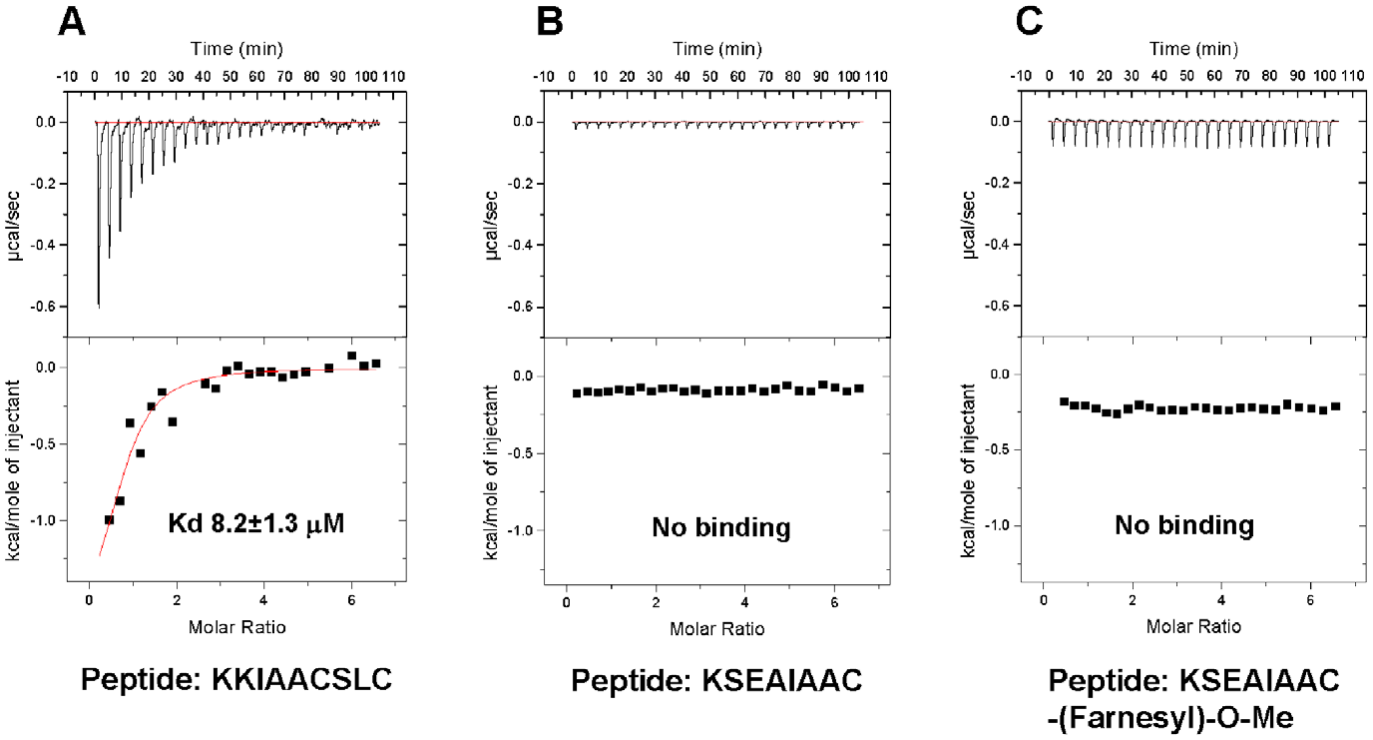
Isothermal titration calorimetric analysis of the binding of a C-terminal peptide from IP to the PDZ1 domain of PDZK1. Recombinant wild-type PDZ1 domain (0.03 mM in 150 mM NaCl, 0.5 mM tris (2-carboxyethyl) phosphine, 25 mM Tris-pH 8.0) were placed in the titration cell and equilibrated at 20°C. A solution containing 1.0 mM of (A) the C-terminal nonapeptide from IP, KK^411^IAACSLC^417^, or (B) the octapeptide K^407^SEAIAAC^414^ corresponding to the C-terminal sequence of IP from which the last three amino acid –SLC are absent, or (C) the farnesylated-carboxy-methylated form of the same peptide (K^407^SEAIAAC^414)^ were injected in 10 µl aliquots with an interval of 4 minutes between each addition to permit re-equilibration. Titration curves were analyzed and K_d_ values determined using ORIGIN 7.0 software.

**Figure 3:**
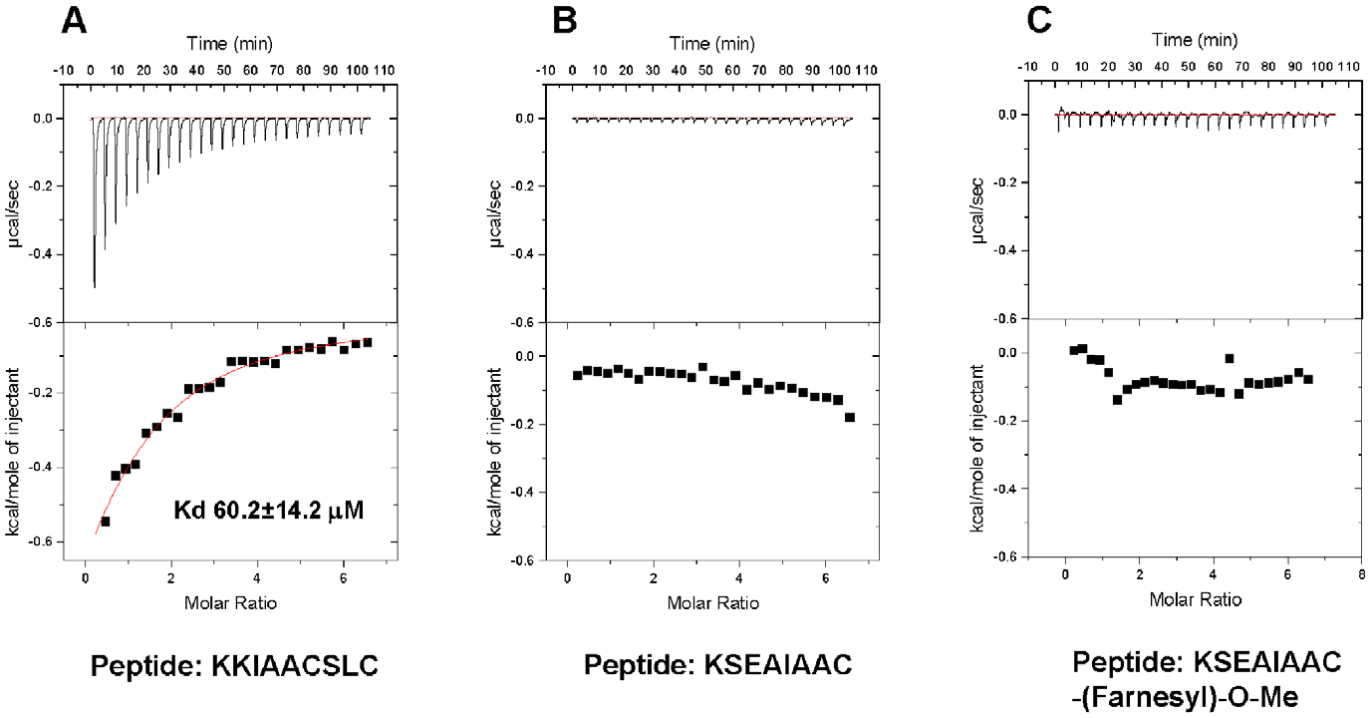
Isothermal titration calorimetric analysis of the binding of a C-terminal peptide from IP to full-length PDZK1. Recombinant full-length PDZK1 (A–C) proteins (0.03 mM in 150 mM NaCl, 0.5 mM tris (2-carboxyethyl) phosphine, 25 mM Tris-pH 8.0) were placed in the titration cell and equilibrated at 20°C. A solution containing 1.0 mM of (A) the C-terminal nonapeptide from IP, KK^411^IAACSLC^417^, or (B) the octapeptide K^407^SEAIAAC^414^ corresponding to the C-terminal sequence of IP from which the last three amino acid –SLC are absent, or (C) the farnesylated-carboxy-methylated form of the same peptide (K^407^SEAIAAC^414)^ were injected in 10 µl aliquots with an interval of 4 minutes between each addition to permit re-equilibration. Titration curves were analyzed and K_d_ values determined using ORIGIN 7.0 software.

Binding of peptides 1–3 to a recombinant protein corresponding to PDZ domain 1 (PDZ1) and to full length PDZK1 was then evaluated by ITC. These experiments revealed the presence of a high affinity binding site for the IP (KK^411^IAACSLC^417^) carboxy-terminal peptide on PDZ1 (K_d_ of 8.2±1.3 µM) (Fig. 2A), but no detectable binding for either the second peptide (K^407^SEAIAAC^414^) (Fig. 2B) or its farnesylated, carboxymethylated form (K^407^SEAIAAC^414^-(Farnesyl)-O-Me) was observed (Fig. 2C). Moreover, peptide 1, but not peptides 2 or 3, also specifically bound to full length PDZK1 (Fig. 3A–3C), but with a lower binding affinity for PDZK1 (K_d_ of 60.2±14.2 µM) than that for the PDZ1 isolated domain (Fig. 2A–2C). This could be explained by a decreased access of the IP peptide to both PDZ1 and PDZ3 because of the ability of PDZ3 to trigger dimerization of PDZK1 and the ability of the carboxy terminal tail of PDZK1 (-TEM) to interact with PDZ1 [32], [33], [34].

### Crystal Structure of PDZK1 PDZ1 with Bound IP Target Peptide

**Figure 4:**
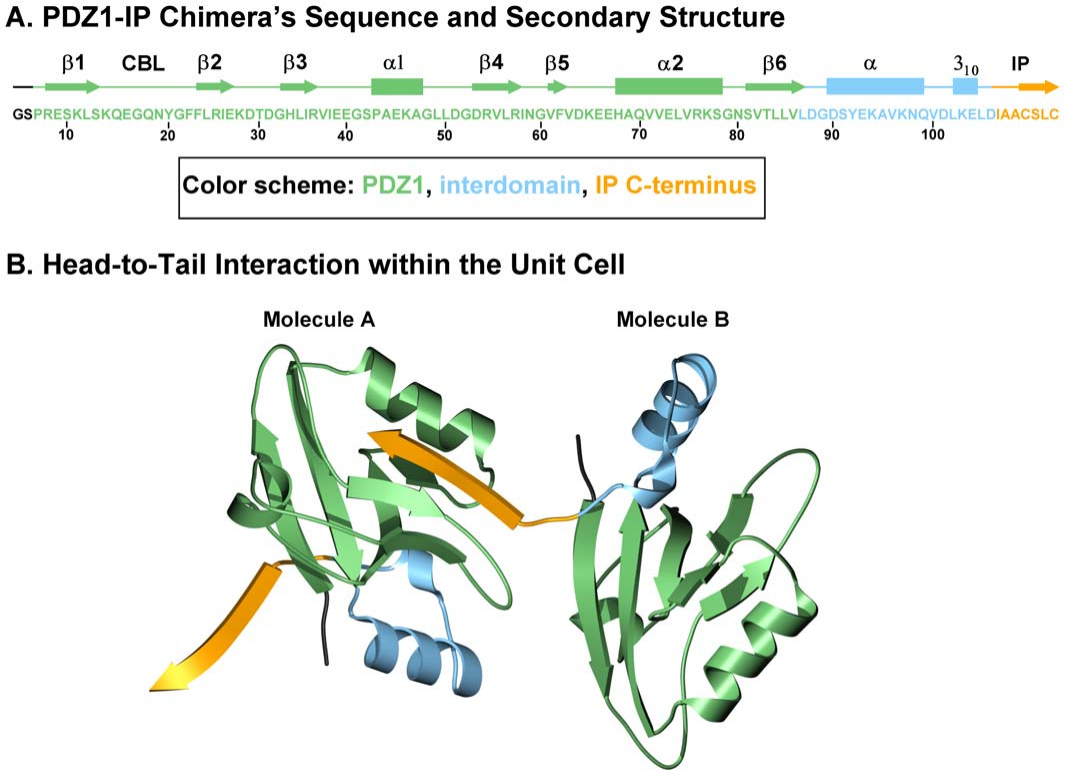
X-ray crystal structure of the PDZ1-IP target peptide chimera. **A**: Amino acid sequence of the recombinant chimeric protein used for crystallization: N-terminal Gly-Ser dipeptide derived from the cloning vector (black), PDZ1 domain (residues 7–86 (PDZK1 numbering), green), partial interdomain segment (87–106, lies between the PDZ1 and PDZ2 domains, blue) and seven carboxy-terminal residues of IP (^411^IAACSLC^417^, IP numbering, yellow). Regions of secondary structure (β strands and α helices) and the carboxylate-binding loop (CBL) are indicated above the sequence. **B**: Asymmetric unit showing the head-to-tail arrangement of two chimeric molecules. This figure was generated using POVScript [47] using the color scheme in panel A.

**Figure 5:**
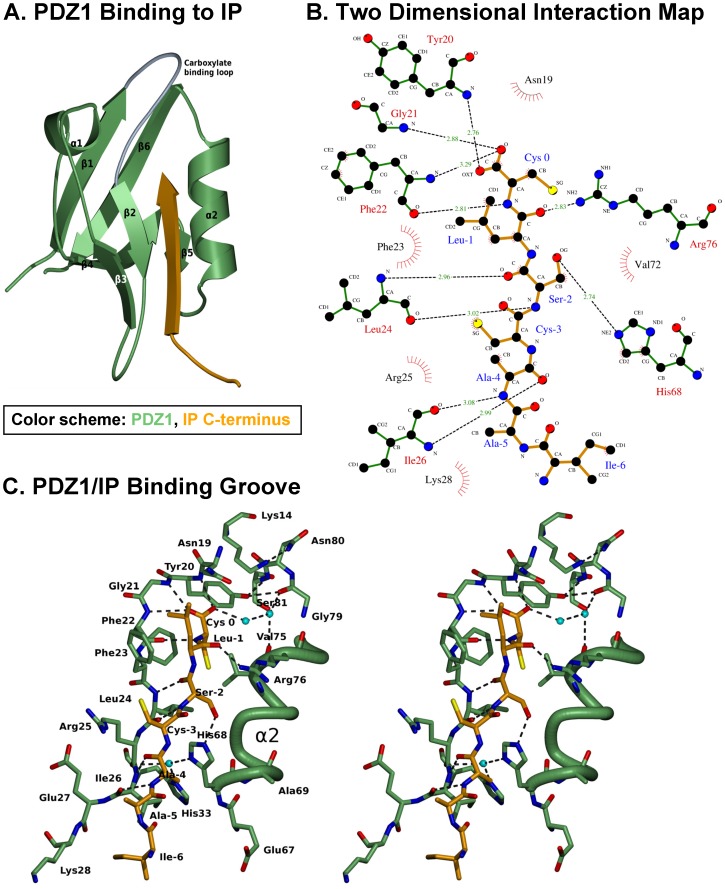
Structure of the C-terminal IP target peptide binding to PDZ1. **A**: Ribbon diagram showing the three dimensional structure of PDZ1 (residues 7–86, green with gray carboxylate-binding loop) and the bound C-terminus of IP (^−6^IAACSLC^0^, yellow) from an adjacent molecule in the asymmetric unit. Six β-strands (β1-β6), two α-helices (α1–α2) and carboxylate-binding loop (dark gray) are indicated. Vector derived residues have been omitted for clarity. **B**: Two-dimensional representation of interactions between PDZ1 (green) and the C-terminal IP target peptide (yellow). Hydrogen bonds are shown as dashed lines and hydrophobic interactions as arcs with radial spokes. This figure was generated using LIGPLOT [48]. **C**: Stereo representation of the ligand-binding groove of PDZ1 (green) and the IP target peptide (yellow). Oxygen, nitrogen and waters molecules are shown in red, dark blue and cyan, respectively. Sulfur atoms are colored in yellow. Hydrogen bonds are shown as dashed lines. The orientation is similar to that in panel A.

As stated, PDZK1 is a multi-PDZ domain protein containing four PDZ domains (PDZ1– PDZ4). While the crystal structure of numerous individual PDZ domains, either alone or in complex with target ‘PDZ ligand(s)’, have been reported [27], [32], [35], to the best of our knowledge the complete structure of a protein containing two or more PDZ domains has not been solved to date. Hence, our strategy to study the structure of PDZK1 in complex with the target ‘PDZ ligand’ of the IP was to crystallize and analyze the structure of a chimeric recombinant protein incorporating the PDZ1 domain of PDZK1 fused to the C-terminal 7 amino acid residues of the mouse IP (Fig. 1). This approach is based on that previously reported for the structural analyses of human NHERF PDZ1 bound to the cystic fibrosis transmembrane conductance regulator (CFTR) [36], the β_2_ adrenergic and platelet-derived growth factor receptors [37] and the interaction of the HDL receptor, scavenger receptor class B type I (SR-BI) with the PDZ1 [27] and PDZ3 [32] domains of PDZK1. We generated a chimeric recombinant protein (sequence in Fig. 4A) for structural analysis. This protein comprises an N-terminal glycine and serine dipeptide (encoded by the cloning vector, black in Fig. 4A), residues 7–106 of PDZK1, including the first PDZ domain of PDZK1 (residues 7–86, green) and 20 residues (87–106, blue) from the 47 residue segment that lies between the PDZ1 and PDZ2 domains (interdomain) whose C-terminus was extended by addition of the seven carboxy-terminal residues of its target peptide on IP (yellow): ^411^IAACSLC^417^. In Fig. 5, the alternative numbering scheme ^−6^IAACSLC^ 0^ (numbering from the C-terminal residue “0”) which is commonly used for PDZ domain targeted peptide sequences is employed and will distinguish the target peptide residues of the PDZ ligand from those of PDZK1. We grew crystals, collected X-ray diffraction data at the NSLS (Brookhaven National Laboratory) and solved the crystal structure at 1.70 Å resolution (Table 1) by molecular replacement using the coordinates of the crystal structure of the first PDZ domain of PDZK1 bound to the SR-BI carboxy-terminus as the search model [27]. The high quality of the electron density map permitted unequivocal assignment of all amino acid side chains. Fig. 4B shows that in the crystal, the IP peptide (yellow) of one molecule (B) interacts with the peptide binding pocket of the PDZ domain (green) of an adjacent molecule (A) in the asymmetric unit, resulting in an “infinite chain” of head-to-tail molecules. The interdomain sequence is shown in blue. Molecules A and B in the asymmetric unit have similar structures and superimpose with a root-mean-square deviation of 0.68 Å over all main chain atoms (or 0.97 Å over all atoms) between ^7^Pro and ^113^Cys. Molecule B was judged to have a superior quality electron density and will therefore be used to describe the structure below. For clarity, we numbered the residues in the PDZ1 portion of the structure to correspond to the numbering of this domain in the intact murine PDZK1 sequence. Figure 5A shows the PDZ1 domain (residues 7–86 in green with the carboxylate-binding loop (CBL) highlighted in gray, the N- and C-terminal extensions have been removed for clarity) and the bound IP peptide (yellow) from an adjacent molecule. The tertiary structure of PDZ1 matches that previously described [27] – a compact globular structure containing a six-stranded anti-parallel β-barrel (β1–β6) flanked by two α-helices (α1 and α2) [35], [38]. An additional α-helix and a 3_10_ helix are found in the portion of the interdomain sequence included in the structure (Fig. 4A and 4B, not shown in Fig. 5). To facilitate the description of the structure, we will designate the secondary structural elements (e.g., β1, α2, CBL) in parenthesis following residues that are in those elements (e.g., ^21^Gly (CBL)). As has been described for target peptide binding to other PDZ domains [27], [32], [35], [37], [39], the C-terminus of IP (^−6^IAACSLC^ 0^) inserts into a groove formed between the β2 strand and the α2 helix, and the CBL formed by ^14^Lys… ^20^Tyr-^21^Gly-^22^Phe-^23^Phe-^24^Leu that connects the β1 and β2 strands. The backbone amides and carbonyls of the target peptide form classic anti-parallel β-sheet hydrogen bonding interactions beginning with carbonyl of ^22^Phe of the CBL and extending through β2 from ^23^Phe to ^26^Ile (Figs. 5B and 5C).

**Figure 6:**
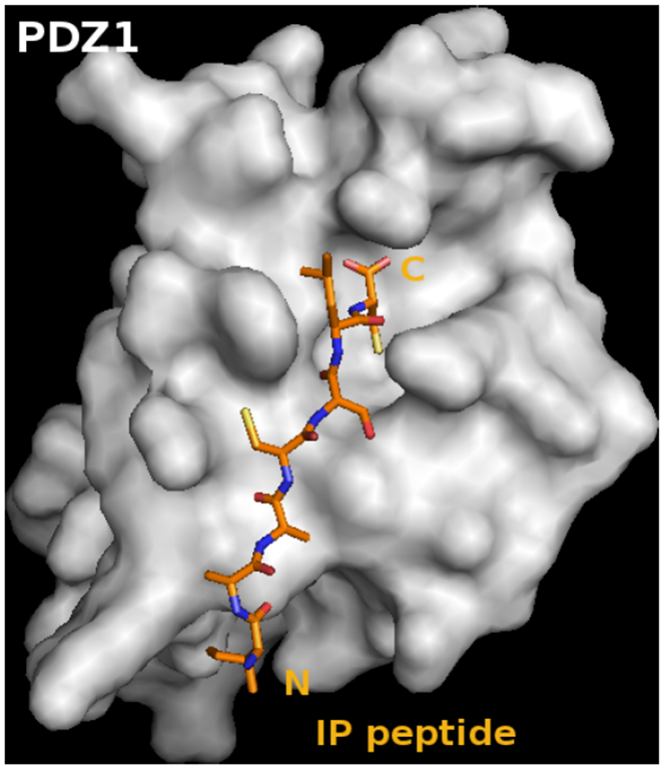
Surface topology of PDZ1 and its bound target peptide. Surfaces of PDZ1 (gray) is shown bound to the IP target peptide (^−6^IAACSLC^0^, stick representation with the amino and carboxy-termini labeled “N” and “C”). The hydrophobic binding pocket accommodating the side chain of ^0^Cys can be seen as a deep cavity near the C-terminus of the target peptide. This figure was generated using PyMOL [49].

There is a well-defined, extensive, hydrogen bonding network directly and indirectly connecting the target peptide, multiple regions of PDZ1 and bound water molecules (dashed lines in Fig. 5B and 5C). The carboxylate group of the target peptide’s ^0^Cys makes hydrogen bonds with the amide nitrogens of ^20^Tyr, ^21^Gly and ^22^Phe in the CBL and, through water-mediated interactions, the carbonyls of ^75^Val and ^76^Arg of α2 and the carbonyl of ^79^Gly. In addition, the hydroxyl group of ^20^Tyr (CBL) forms hydrogen bonds with the carbonyl of ^79^Gly in α2 and the amide of ^81^Ser in β6, interconnecting multiple secondary structure elements of PDZ1. The amide nitrogen of ^0^Cys makes a hydrogen bond to the carbonyl oxygen of ^22^Phe (CBL) and the side chain fits into a deep hydrophobic pocket composed of the side chains of ^22^Phe and ^24^Leu of the CBL and ^72^Val, ^75^Val and ^76^Arg of α2 (Fig. 5B and 5C)). There are also hydrogen bonds between the carbonyl oxygen of ^−1^Leu and the side chain of ^76^Arg (α2), the side chain of ^−2^Ser and the side chain of ^68^His (α2), the amide nitrogen of ^−2^Ser and the carbonyl oxygen of ^24^Leu, the carbonyl of ^−2^Ser and the amide of ^24^Leu, the amide nitrogen of ^26^Ile and the carbonyl oxygen of ^−4^Ala and the amide of ^−4^Ala and the carbonyl oxygen of ^26^Ile (Fig. 5B and 5C). There are a number of hydrophobic interactions between the IP peptide and PDZ1: ^19^Asn and ^22^Phe (CBL) with ^0^Cys, ^23^Phe (CBL) with ^−1^Leu, ^23^Phe (CBL) and ^72^Val (α2) with ^−2^Ser, ^25^Arg (β2) with ^−3^Cys, ^68^His (α2) with ^−4^Ala and ^28^Lys with ^−6^Ile (Fig. 4B). The surface topology of the structure demonstrates that IP’s carboxy-terminal peptide fits in the deep groove within PDZ1 (Fig. 6), showing a similar shape to that described for the SR-BI/PDZ1 interaction [32].

The structure provides important clues for the understanding of the ITC data and highlights the role of IP’s three carboxy-terminal amino acids (-SLC) in particular for the stability of the interaction.

## Discussion

PDZK1 is a multi-PDZ domain-containing adaptor protein known to play a role in the expression, localization and function of several cell surface receptors and ion channels [8]. One of its molecular interactants is the prostacyclin receptor (IP), whose interaction with PDZK1 was recently described [19]. Through those studies involving yeast-two-hybrid- and co-immunoprecipitation-type studies in mammalian cells, the IP was found to interact with PDZ1, PDZ3 and PDZ4, but not PDZ2 of PDZK1. These interactions involve a Class I type of binding typically seen for protein interactants with carboxy-terminal Ser/Thr in position -2 and a hydrophobic amino acid residue in position 0 (Ser/Thr–X–Φ–COOH) of the ‘PDZ ligand’ of the target interacting protein [19]. Functionally, the interaction between PDZK1 and IP appears to be essential for the role of prostacyclin in re-endothelialisation, promoting endothelial cell migration and angiogenesis [19]. Herein, the detailed *in vitro* molecular analysis of the interaction between IP and the first PDZ domain of PDZK1 (PDZ1) revealed that only the non-isoprenylated form of the carboxy-terminus of IP is capable of interacting with PDZ1 suggesting a alternative role for the isoprenylated form, presumably anchoring IP in the plasma membrane [20]. As stated, our previous cell-based studies suggested that the interaction of the IP with PDZK1 is independent of the isoprenylation status of the IP [19], while the current biophysical study investigating the interaction of peptides based on the C-terminal regions of the IP demonstrates that neither PDZK1 nor its PDZ1 bind the isoprenylated form of the IP sequence, *in vitro* at least. The basis of the apparent inconsistency between the two studies is currently unknown but may possibly be due to differences between the experimental approaches used and is most likely due to unexplained differences between that which occurs *in vitro* and *in vivo* in the cell. The binding affinity (K_d_, 8.2±1.3 µM) of peptide 1 corresponding to the PDZ ligand of the IP to interact with PDZ1 *in vitro* appears to be in the same order as that of SR-BI binding to PDZ1 (K_d_, 2.6 µM) of PDZK1 [27]. Furthermore, while the non-isoprenylated form of the IP also specifically bound to full length PDZK1, incorporating PDZ1– PDZ4, it did so but with a lower binding affinity for PDZK1 (K_d_, 60.2±14.2 µM) than that for PDZ1 alone. These findings are consistent with the ability of the IP to bind to multiple PDZ domains within the full length PDZK1 itself [19] and with experimental observations from multi-PDZ domain containing proteins whereby intradomain and/or intramolecular interactions have been proposed to regulate PDZ domain binding potential [5], [40], [41], [42]. Indeed, the PDZ3 domain of PDZK1 promotes dimerization of the molecule and the carboxy terminal tail of PDZK1 (-TEM) is able to interact with its PDZ1 domain likely resulting in decreased accessibility of both PDZ1 and PDZ3 for the IP peptide [32], [33], [34].

In addition, these findings further contribute to the evidence that the binding for PDZ ligands within full-length multiple PDZ domain-containing proteins can differ substantially from that of the individual PDZ domains in isolation [43], [44], [45]. Moreover, in many cases, the PDZ domains of such multiple PDZ domain-containing proteins are grouped, including in pairs (e.g. PDZ1/2 of post-synaptic density 95 (PSD-95)) and triplets (e.g. PDZ1–3 and PDZ4–6 of glutamate receptor-interacting protein (GRIP)). While the significance of this grouping is unclear, there is evidence to suggest that multiple PDZ domains can co-operate to mediate binding to target PDZ ligands. For example, the PDZ2 of syntenin binds syndecan, neurexin, and ephrin-B1 only when paired with PDZ1, and does not interact when presented in isolation [43]. In addition, it has been suggested that one PDZ domain may influence the folding of an adjacent PDZ domain. For example, PDZ5 of GRIP alone is unstructured in solution and fails to bind GluR2 [46]. However, when presented in tandem with PDZ4, PDZ5 becomes highly structured and GluR2 binding is restored [46]. In time, resolving the crystal structure of the ‘PDZ ligand’ of the IP with some or all of the other PDZ domains of PDZK1, including consideration of the capability of whether peptides 1, 2 or 3 can bind and/or, when possible, where two or more/multiple PDZ domains are present should provide further insight into the interaction of the IP with the multi-functional protein PDZK1.

We recently solved the structures of the interactions between the HDL receptor SR-BI with the first and with the third PDZ domains of PDZK1 by X-ray crystallography [27], [32]. The comparison of both crystal structures provided important findings to determine the molecular nature of the interactions, define the amino acids of both proteins involved and also allowed understanding as to why one of the interactions was high affinity (SR-BI/PDZ1), while the other lower affinity (SR-BI/PDZ3). The crystal structure described herein, analyzing the interaction between the carboxy-terminus of the IP and PDZ1 highlights the plasticity of a given PDZ domain to interact with the carboxy-termini of binding targets with very different amino acid compositions: -IAACSLC for IP versus -VLQEAKL for SR-BI. A close look at the nature of the interaction reveals several differences between the IP/PDZ1 and SR-BI/PDZ1 structures (Fig. 5) [27]. The side chain of ^20^Tyr, which played a major role in the SR-BI/PDZ1 and SR-BI/PDZ3 interactions, using a complex hydrogen bond-water molecule network between the hydroxyl group of ^20^Tyr and the carboxy-terminal last amino acid (^0^Leu) of SR-BI, is not participating in the interaction with the carboxy-terminal last amino acid (^0^Cys) of IP. ^68^His (α2), which did not participate in the SR-BI/PDZ1 interaction appears to play an important role in the IP/PDZ1 interaction, forming an hydrogen bond with the side chain of ^−2^Ser. There is a hydrophobic interaction between ^19^Asn and ^0^Cys in the IP/PDZ1 interaction which did not exist in the SR-BI/PDZ1 interaction. However, ^76^Arg does not appear to form a hydrophobic interaction with the IP carboxy-terminal peptide, while such interaction was taking place with the carboxy-terminal leucine (^0^Leu) of SR-BI. The structure also highlights the importance of the last three carboxy-terminal amino acids of IP, responsible for the majority of hydrogen bonds formation and hydrophobic interactions between IP and PDZ1, a function that could not be assumed by a farnesyl group (Fig. 4 and 5).

Collectively, these findings highlight the importance of the information gathered from the crystal structure and may serve as the basis for the identification of new compounds, such as peptidomimetics, that may either selectively stabilize or disrupt the IP/PDZ1 interaction, with the ultimate goal of designing new therapeutic modalities for the treatment of some of the cardiovascular disease etiologies in which prostacyclin and its receptor, the IP, are most widely implicated.
